# The investigation and prevalence of pulmonary embolism among emergency department patients with acute exacerbations of chronic obstructive pulmonary disease (AECOPD): A multi-centered linked administrative database study

**DOI:** 10.1371/journal.pone.0340308

**Published:** 2026-01-08

**Authors:** Brian H. Rowe, Esther H. Yang, Cristina Villa-Roel, Bo Zheng, Irvin Mayers

**Affiliations:** 1 Department of Emergency Medicine, University of Alberta, Edmonton, Alberta, Canada; 2 Provincial Research Data Services, Alberta Health Services, Edmonton, Alberta, Canada; 3 Department of Medicine, University of Alberta, Edmonton, Alberta, Canada; Al Nasiriyah Teaching Hospital, IRAQ

## Abstract

**Background:**

Although patients with acute exacerbation of chronic obstructive pulmonary disease (AECOPD) may be investigated for pulmonary embolism (PE) in the emergency department (ED), little is known about the prevalence of PE and factors associated with investigation. We sought to evaluate the PE prevalence among patients presenting to the ED with AECOPD.

**Methods:**

All adult patients presenting with AECOPD to six EDs between January 2015 and June 2021 using ICD-10-CA codes from administrative data. The primary outcomes were the investigation for and prevalence of PE. Conventional, age-adjusted D-dimer (AADD) and chest imaging are reported. A multivariable logistic regression was used to identify predictors of investigations for PE among patients with AECOPD, including demographic characteristics, comorbidities, and ED presentation data as covariates.

**Results:**

Of the 25,510 patients with AECOPD, 12,164 (48%) patients (median age 70 years, 50% males, 46% hospitalized) were included after applying exclusion criteria. Overall, 2,072 (17%) patients received at least one test for PE: 84% had a D-dimer, 44% had a chest CT and 2% had lung scans. Overall, 68 (0.5%) patients received a diagnosis of PE; 41 (0.3%) received a PE co-diagnosis in the ED and 27 (0.2%) patients received a primary PE diagnosis while hospitalized. Use of an AADD could reduce CT image ordering by approximately 13%. Overall, 852 (7%) returned to the ED and 490 (4%) died within 30 days. The presence of chest pain (aOR=2.71; 95% CI: 2.24–3.28) and cough/congestion (aOR=0.57; 95% CI: 0.46–0.70) increased and decreased PE investigations, respectively.

**Conclusion:**

The overall prevalence of PE among patients presenting to the ED with AECOPD was low (less than 1%). While acknowledging PE may occur concurrently with AECOPD, clinicians should be cautious to avoid over-investigation, which has a negative impact on operational flow, increases costs, and may be harmful to patients. Evidence-based pathways using information readily available at presentation and selective investigations (e.g., decision rules and AADD cut-offs) have the potential to improve resource use and facilitate shared decision-making in the acute setting.

## Introduction

Chronic obstructive pulmonary disease (COPD) is a common, chronic lung disease characterized by progressive and not fully reversible airflow limitation [1]. Persistent worsening in patients’ baseline symptoms of dyspnea, cough and sputum production (quantity and purulence) are required to define an acute exacerbation of COPD (AECOPD). As the disease progresses, exacerbations become more frequent and severe [2]. Exacerbations often result in emergency department (ED) presentations and when severe, require hospitalization. In fact, in patients with COPD, the disease itself is the single most important determinant of overall health outcomes [3], as it is associated with increased morbidity, substantial ED visits, hospital admission and death [4–6].

Exacerbations occur due to a variety of reasons including, but not limited to viral and bacterial infections, environmental pollution, and medication non-adherence. In addition, patients with AECOPD can have complications such as heart failure, pneumonia, arrythmias and pulmonary embolism (PE). The standard ED management of COPD exacerbations consists of complete blood count, electrolyte and renal function panels, simple chest radiography, electrocardiography, and selective cardiorespiratory biomarkers (e.g., D-dimer, troponin, and/or brain natriuretic peptide [BNP]). Debate exists over the frequency of PE in this patient group and how to investigate for the condition in the ED management of AECOPD [7].

A recent systematic review and meta-analysis found that approximately 12% (95% confidence interval [CI]: 9% to 16%) of patients with AECOPD had an underlying PE [7]. Among the included studies, only one study included patients from the ED and found a lower prevalence of PE during AECOPD (3.3%) [8]; however, other ED-based studies have identified over-testing for PE and low PE rates in ED patients [9]. Reviews have showed that the prevalence of PE was significantly higher among patients who required hospitalization for AECOPD; however, the prevalence was also highly variable, ranging from 2% to 33% across studies. This variability raises concerns about potentially missed cases of PE and suggests that clinicians should consider the possibility of PE in patients presenting to the ED with AECOPD.

Most currently available studies may not reflect the actual prevalence of PE during AECOPD in the ED setting [7,10,11]. Additionally, little is known about how often patients experiencing an AECOPD are subjected to PE workups, particularly using pre-test probability scales, appropriate biomarkers, and advanced imaging, including ventilation-perfusion (VQ) lung scans and computerized tomography (CT) to rule-out PE. The goal of this study was to determine the frequency of PE investigation and confirmation, and to explore factors associated with PE investigations for patients presenting to the ED with AECOPD.

## Methods

### Study design and population

This project was a secondary analysis of a population-based cohort study of all adults (≥18 years) who presented to six EDs in Edmonton, Alberta and were diagnosed with AECOPD between January 1, 2015 and June 30, 2021. Patients with a co-diagnosis of acute myocardial infarction or acute coronary syndrome, those who had died prior to ED presentation, and patients who left without being seen by an ED physician were excluded from our analyses. In this study, a diagnosis of PE required imaging confirmation. Therefore, those with co-diagnosis of PE in the ED without any D-dimer testing or/and advanced chest imaging were also excluded. This ensures that the study population consists of patients whose PE diagnosis was confirmed during their index ED visit. Only first index visits were considered in cases of patients with multiple ED visits to avoid counting the same individual multiple times and to make sure each patient contributed a single observation.

### Data sources and variables of interest

De-identified and individual-level databases within the population-based linked health administrative data from Alberta Health Services (AHS) were used to determine the study cohort. The linkage of data was completed using unique identifiers. Once the linkage was complete, all personal identifiers were removed. All datasets are maintained and updated in the AHS Enterprise Data Warehouse.

The study cohort was determined using the National Ambulatory Care Reporting System (NACRS) which reflects all visits to any ED in Alberta. Each NACRS record represents a unique service, and includes a unique identifier, start and end dates and times, basic patient demographics (e.g., age and sex), the Canadian Triage and Acuity Scale (CTAS) score at ED presentation, disposition status, and diagnostic fields including the main diagnosis and up to nine additional diagnoses using the *International Classification of Disease, 10*^*th*^ *Revision, Canadian Enhancement* [ICD-10-CA]. NACRS data undergoes chart abstraction by trained health record abstractors following CIHI guidelines, while other information is interfaced directly from electronic medical records. These data sources are routinely used for administrative research purposes in Alberta and across Canada. The data contained within NACRS have been shown to be reliable and valid, and in Alberta, represents approximately 99.9% of all ED visits with limited missing data [12]. The CTAS score is a standard acuity score applied to all Canadian EDs, with a score of 1 representing a patient requiring resuscitation and 5 representing a non-urgent presentation [13].

Each AECOPD case was defined by either the first or second diagnosis field containing the ICD-10 code ‘J44.x’ [14]. Pulmonary embolism was defined by the first three diagnosis fields containing the ICD-10 code ‘I26.x’ during the ED encounter or a subsequent hospitalization, alongside a primary or secondary diagnosis of AECOPD. Presenting complaints and requests for consultation for ED visits were collected through the Emergency Department Information Tracking System (EDIS). The provincial laboratory databases capture all general laboratory tests performed across the province and were used to identify those who underwent D-dimer and complete blood count (CBC) testing within the index ED visit. For this study, the D-dimer test results were classified as positive using the conventional (500 μg/L) without adjusting for patient age. In addition, we also calculated the age-adjusted D-dimer (AADD) and estimated how many patients qualified for further testing assuming these cutoffs had been applied (by age x 10 μg/L in patients 50 years or older). The provincial diagnostic imaging database captures all imaging performed across the province within AHS facilities and was used to identify those who underwent simple and advanced imaging (e.g., chest x-ray, chest CT, and VQ lung scans) to evaluate for PE during their ED visit. Provincial Registry data and Alberta Vital Statistics data were used to define death within 30 days of the index ED visit. Discharge Abstract Data (DAD), NACRS, and Practitioners Claims were used to define patients’ comorbidities using ICD-10 and ICD-9 codes in two years prior to their first index ED visit.

### Outcomes

The primary outcome was the prevalence of PE in the index ED visit or during hospitalization among patients with AECOPD. If a PE code was recorded during their ED visit, it was assumed to be identified during the index ED visit. If the PE code was not included in the ED, it was assumed to be identified during that hospitalization. Secondary outcomes included several operational and clinical outcomes, such as disposition status (e.g., admission or discharge), ED length of stay (LOS), revisit within 30- and 180- days of the index ED visit, and all-cause death within 30 days.

### Statistical analysis

Descriptive data are reported using proportions, means with standard deviations (SD), or medians with interquartile range (IQR), as appropriate. Outcomes were compared between patients with and without testing for PE using Pearson’s χ^2^ test for categorical variables, Student’s *t*-test for normally distributed continuous variables, and Mann-Whitney test for non-normally distributed continuous variables.

A multivariable logistic regression including age, sex, CTAS score, mode and time of arrival, presenting complaint, and comorbidities was used to explore the factors associated with investigations for PE. Unadjusted and adjusted odds ratios (ORs) with the corresponding 95% CIs were reported. In addition, a Kaplan-Meier survival curve was generated to examine deaths at 30 days between those who underwent investigations for PE and those who did not. Censoring was applied to patients who were alive at the end of the 30-day period. All analyses were conducted using SAS, version 9.4 (SAS Institute, Cary, NC, USA) and a p-value <0.05 was considered statistically significant.

### Ethics

The study protocol was approved by the University of Alberta Heath Research Ethics Board – Health Panel 3 (Pro00097180), with a waiver of individual informed consent. Written informed consent was not obtained from any patient due to minimal risk associated with accessing the administrative database. Operational, administrative and data sharing agreements were obtained between the research team and AHS (the data custodian). Patients and providers were unaware of the study at the time of presentation.

## Results

### Characteristics of study participants

**Fig 1:**
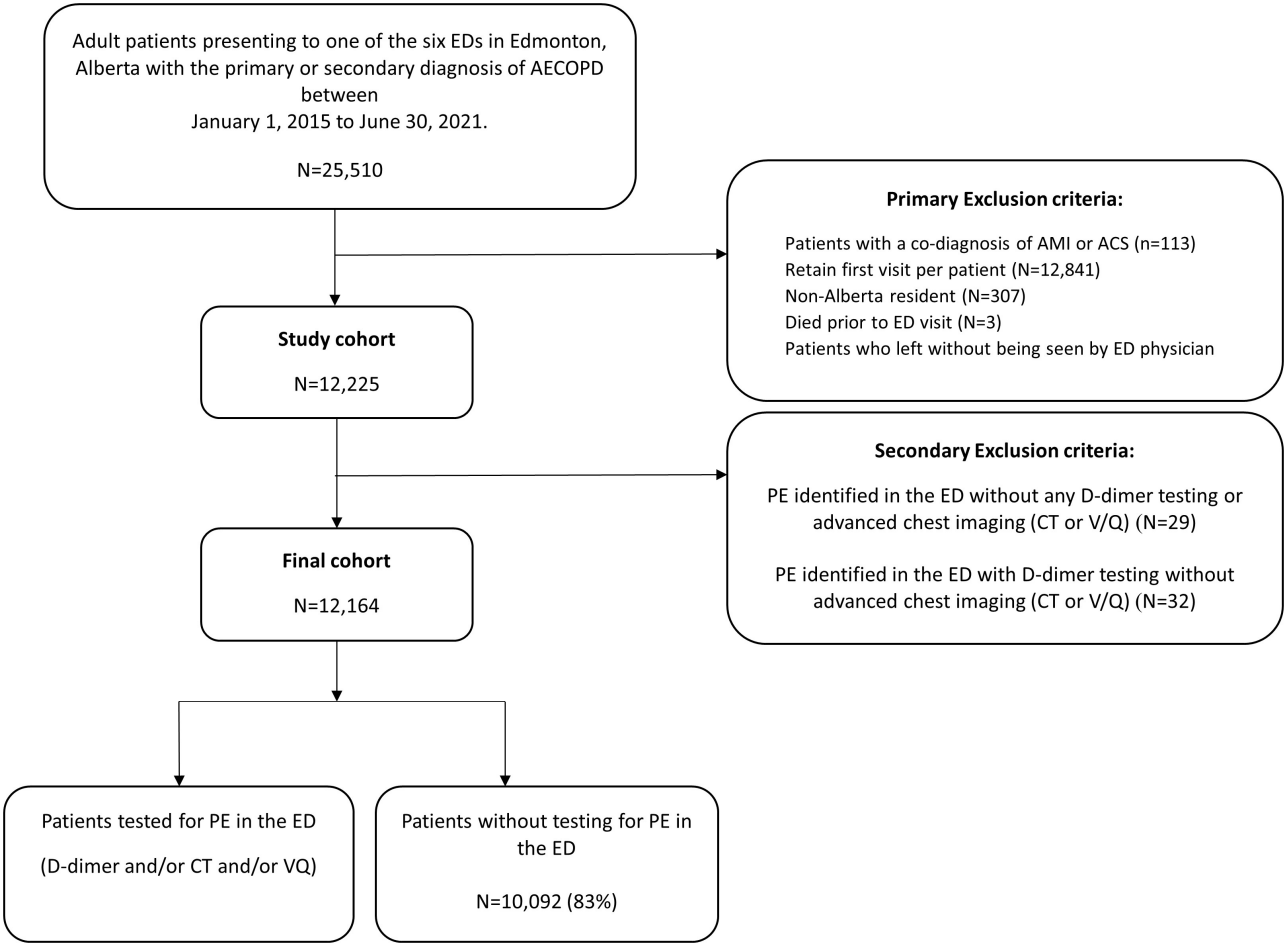
Flow diagram of cohort development. Abbreviations: AECOPD = acute exacerbation of COPD; CT = computer tomography; ED = Emergency department; PE = pulmonary embolism; V/Q = Ventilation-perfusion lung scan; AMI = acute myocardial infarction; ACS = acute coronary syndrome.

Of the 25,510 patients with a primary or secondary ED diagnosis of AECOPD, 12,164 (48%) were included over the 6-year study period after applying exclusion criteria; 2,072 (17%) patients underwent further investigations for PE (Fig 1). Characteristics at presentation are reported in Table 1. The median age was 70 years (IQR: 60, 79) and 50.4% were male. Shortness of breath (60%) was the most common presenting complaint, and most patients received chest radiography and CBC testing in the ED. Among those who underwent further investigations documented for PE, 83.7% received D-dimer testing, 44.1% received a chest CT scan and 2.4% received a VQ lung scan. In addition, there was significant variation observed in the proportion of patients with AECOPD receiving at least one test for PE across sites, from a low of 11.8% at a community site to a high of 19.8% at a teaching site (p < 0.0001).

**Table 1:** Characteristics of 12,164 patients presenting to emergency departments with an acute exacerbation of chronic obstructive pulmonary disease based on their investigations for pulmonary embolism.

	Total N = 12,164	Patients not tested for PE N = 10,092	Patients tested for PE N = 2,072
**Age (years), median (IQR)**	70 (60, 79)	70 (61, 80)	68 (60, 77)
**Male sex (n {%})**	6128 (50.4)	5077 (50.3)	1051 (50.7)
**Triage code (n {%})**			
1/ 2	4980 (40.9)	3996 (39.6)	984 (47.5)
3	6575 (54.1)	5554 (55.0)	1021 (49.3)
4/ 5	608 (5.0)	541 (5.4)	67 (3.2)
Missing	1 (0.0)	1 (0.0)	0 (0.0)
**Mode of Arrival (n {%})**			
EMS arrival	5653 (46.5)	4783 (47.4)	870 (42.0)
**Time of day (n {%})**			
Daytime (08:01–16:00)	6160 (50.7)	5038 (49.9)	1122 (54.2)
Evening (16:01–24:00)	4064 (33.4)	3424 (33.9)	640 (30.9)
Night (00:01–08:00)	1939 (15.9)	1630 (16.2)	309 (14.9)
**Presenting complaints**	12164	10092	2072
Shortness of breath	7272 (59.8)	5961 (59.1)	1311 (63.3)
Cough/congestion	1763 (14.5)	1607 (15.9)	156 (7.5)
Chest pain (Cardiac features)/CTAS 2	562 (4.6)	363 (3.6)	199 (9.6)
Chest pain (Non-cardiac features)/CTAS 3	353 (2.9)	249 (2.5)	104 (5.0)
Any chest pain	919 (7.6)	615 (6.1)	304 (14.7)
**Comorbidities**			
Hypertension	6229 (51.2)	5206 (51.6)	1023 (49.4)
Diabetes mellitus	3097 (25.5)	2592 (25.7)	505 (24.4)
Coronary artery disease	2336 (19.2)	1965 (19.5)	371 (17.9)
Congestive heart failure	2252 (18.5)	1952 (19.3)	300 (14.5)
Cancer	1662 (13.7)	1326 (13.1)	336 (16.2)
Renal disease	1075 (8.8)	935 (9.3)	140 (6.8)
Venous thromboembolism	422 (3.5)	318 (3.2)	104 (5.0)
**Charlson score, median (IQR)**	2 (1, 3)	2 (1, 3)	2 (1, 3)
**Investigations (n {%})**			
Chest X-ray	11163 (91.8)	9226 (91.4)	1937 (93.5)
CBC	10636 (87.4)	8615 (85.4)	2021 (97.5)
Troponin testing	8592 (70.6)	6719 (66.6)	1873 (90.4)
**Investigations for PE (n {%})**			
D-dimer	1735 (14.3)	N/A	1735 (83.7)
Chest CT^α^	913 (7.5)	N/A	913 (44.1)
V/Q scan	49 (0.4)	N/A	49 (2.4)
**Co-diagnosis of PE in the ED**	41 (0.3)	N/A	41 (2.0)
**Co-diagnosis of PE in hospital**	27 (0.2)	18 (0.2)	9 (0.4)

αChest CT includes computed tomography pulmonary angiography (CTPA).

Abbreviation: CBC = Complete blood count; CT = computer tomography; ED = Emergency department; IQR = Interquartile range; PE = pulmonary embolism; V/Q = Ventilation-perfusion lung scan.

### Primary outcome

**Fig 2:**
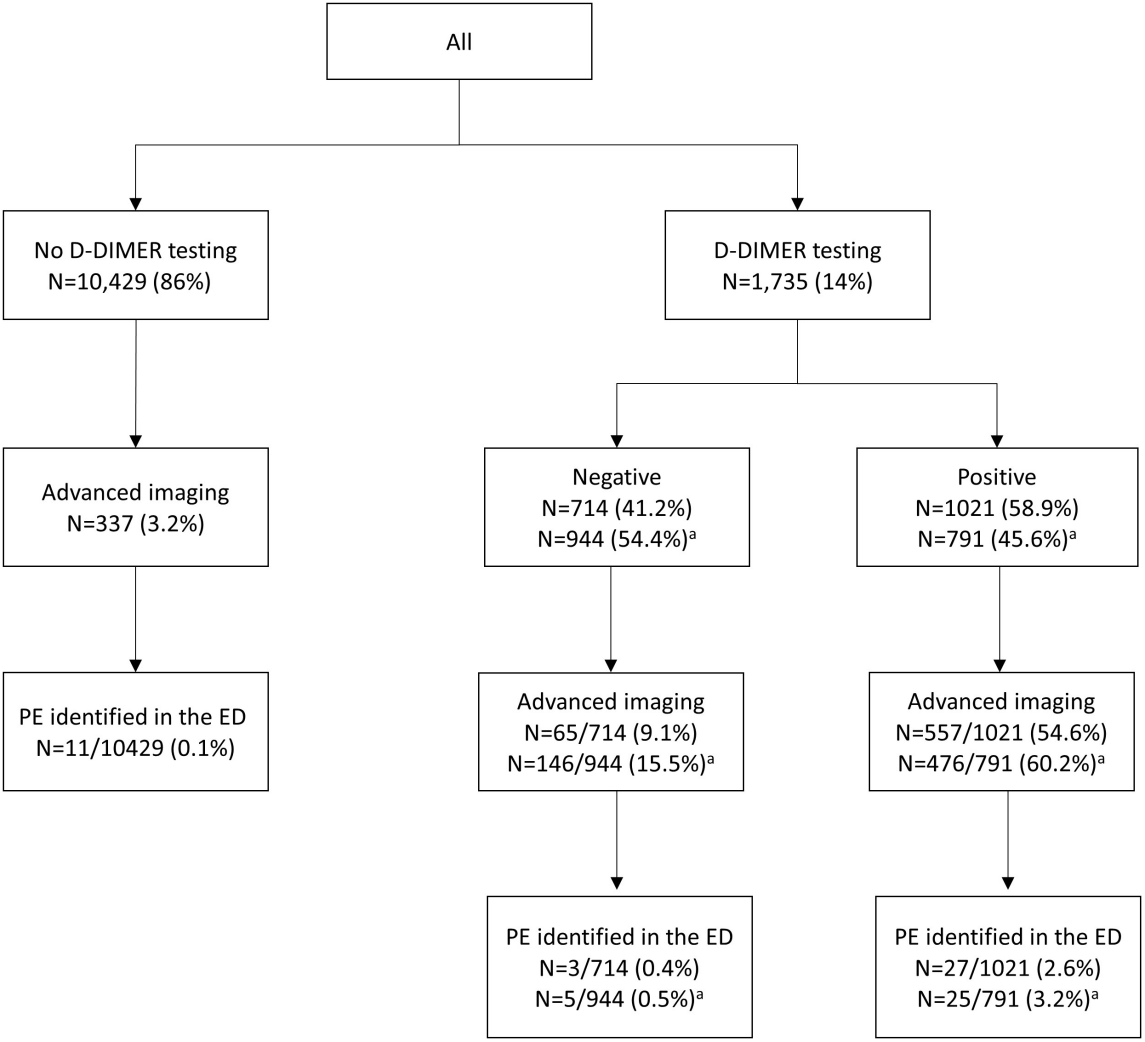
Patients evaluated for pulmonary embolism during their ED visit. a = if age-adjusted D-dimer cut-off employed.

Overall, 68 (0.5%) patients received a diagnosis of PE; 41 (0.3%) received a PE co-diagnosis in the ED and 27 (0.2%) patients received a primary PE diagnosis while hospitalized (Table 1). Of 10,429 patients who did not have D-dimer testing, 337 (3.2%) had either one of the advanced images performed (chest CT scan or VQ lung scan) and 0.1% received a diagnosis of PE in the ED (Fig 2). Of the 1,735 patient who received D-dimer testing, 944 (54%) were classified as negative using the AADD compared to 714 (41%) with the conventional cutoffs (13% increase). Among 944 patients who had a negative AADD result, 16% had further imaging investigations and 0.5% were diagnosed with PE. Among 791 patients who were classified as having a positive AADD, 60% had imaging and 3.2% received a diagnosis of PE.

### Secondary outcomes

As displayed in Table 2, dispositions were similar between those investigated and not investigated for PE. Overall, 46% (5,603) of the patients were admitted. The ED LOS was longer for those investigated than for those not investigated for PE (601 minutes [IQR: 397, 1040] vs. 483 minutes [IQR: 298, 962]; median differences with 95% CI: 117.5 [95.9–139.0]). There were no statistical/clinically relevant differences in LOS among patients who were admitted to the hospital; however, a significant increase in LOS by 113 minutes occurred among patients who were discharged.

**Table 2:** Outcomes of 12,164 patients presenting to emergency departments with an acute exacerbation of chronic obstructive pulmonary disease based on their investigations for pulmonary embolism.

	Total N = 12,164	Patients not tested for PE N = 10,092	Patients tested for PE N = 2,072	p-value or median differences (95% CI)
**Disposition (n {%})**				0.023
Admitted	5603 (46.1)	4642 (46.0)	961 (46.4)	
Discharged	6135 (50.4)	5109 (50.6)	1026 (49.5)	
Transferred	295 (2.4)	226 (2.2)	69 (3.3)	
LAMA	113 (0.9)	100 (1.0)	13 (0.6)	
Died	18 (0.2)	15 (0.2)	3 (0.1)	
**ED PIA in minutes, median (IQR)**	81 (38, 155)	82 (39, 157)	74 (34, 146)	−8.0 (−12.4 to −3.6)
**ED LOS in minutes, median (IQR)**				
Overall	505 (313, 974)	483 (298, 961.5)	601 (397, 1040)	117.5 (95.9 to 139.0)
Admitted	992 (615, 1617)	995 (604, 1622)	978 (666, 1570)	−16.7 (−83.5 to 50.1)
Discharged	342 (243, 469)	325 (232, 443)	438 (321, 576)	113.0 (97.7 to 128.3)
**Return ED within 30 days (n {%})**	852 (7.0)	709 (7.0)	143 (6.9)	0.82
**Return ED visits within 180 days (n {%})** ^**a**^	2270 (18.7)	1904 (18.9)	366 (17.7)	0.20
Number of ED visits, median (IQR)	1 (1, 2)	1 (1, 2)	1 (1, 2)	0.85
PE Investigations, n (%)	394/2255 (17.5)	301/1892 (15.9)	93/363 (25.6)	<0.0001
**Death within 30 days (all-cause) (n {%})**	490 (4.0)	420 (4.2)	70 (3.4)	0.10
**Death during hospital admission after index ED visit (n {%})**	412/5603 (7.2)	356/4642 (7.7)	56/961 (5.8)	0.05
**Death within 30 days when discharged (n {%})**	65/6135 (1.1)	55/5109 (1.1)	10/1026 (1.0)	0.77

^a^Return ED visits within 180 days, inclusive of 30-days of ED return.

Abbreviation: CI = Confidence interval; ED = Emergency department; IQR = Interquartile range; LAMA = Left against medical advice; LOS = Length of stay; OR = Odds ratio; PE = Pulmonary embolism.

Post ED outcomes included 852 (7%) return visits within 30 days, 2,270 (19%) return visits within 180 days, and 490 (4%) deaths within 30 days with no statistically significant differences between those investigated and not investigated for PE (Log-Rank test p = 0.10). Patients who underwent PE investigations were more likely to have the same testing in their next ED visit compared to those who didn’t undergo PE investigations (25.6% vs. 15.9%; p < 0.0001).

### Factors associated with PE investigations

In the multivariable regression model (Table 3), the following factors were significantly associated with PE investigations while in the ED: CTAS scores 1 or 2 (aOR=1.16; 95% CI: 1.04–1.28), presentation with shortness of breath (aOR=1.29; 95% CI: 1.12–1.48) or any chest pain (aOR=2.71; 95% CI: 2.24–3.28), and a history of cancer (aOR = 1.65; 95% CI: 1.40–1.95) or venous thromboembolism (aOR=1.64; 95% CI: 1.30–2.07). Several factors were significantly associated with a lower likelihood of undergoing PE investigations including older age (aOR=0.99; 95% CI: 0.98–0.99), CTAS score 4 or 5 (aOR=0.67; 95% CI: 0.51–0.88), EMS arrival (aOR=0.67; 95% CI: 0.76–0.94), evening presentation (aOR=0.81; 95% CI: 0.73–0.91), presentation with cough/congestion (aOR=0.57; 95% CI: 0.46–0.70), a higher Charlson comorbidity index score (aOR = 0.94; 95% CI: 0.91–0.97) and a history of congestive heart failure (aOR=0.84; 95% CI: 0.73–0.98).

**Table 3:** Factors associated with investigations for pulmonary embolism among patients presenting to emergency departments with an acute exacerbation of chronic obstructive pulmonary disease.

	Unadjusted OR with 95% CI	Adjusted OR with 95% CI
**Male sex**	1.02 (0.93–1.12)	0.97 (0.89–1.08)
**Age**	0.99 (0.98–0.99)	0.99 (0.98–0.99)
**CTAS**		
1/ 2	1.34 (1.22–1.48)	1.16 (1.04–1.28)
3	Ref	Ref
4/ 5	0.67 (0.52–0.88)	0.67 (0.51–0.88)
**EMS arrival**	0.80 (0.73–0.88)	0.85 (0.76–0.94)
**Time of day**		
Daytime (08:01–16:00)	Ref	Ref
Evening (16:01–24:00)	0.84 (0.76–0.93)	0.81 (0.73–0.91)
Night (00:01–08:00)	0.85 (0.74–0.98)	0.84 (0.73–0.97)
**Presenting complaint**		
Shortness of Breath	1.19 (1.08–1.32)	1.29 (1.12–1.48)
Cough/congestion	0.43 (0.36–0.51)	0.57 (0.46–0.70)
Any chest pain	2.65 (2.29–3.07)	2.71 (2.24–3.28)
**Charlson comorbidity index score**	0.96 (0.93–0.98)	0.94 (0.91–0.97)
**Comorbidities**		
Hypertension	0.92 (0.83–1.01)	–
Diabetes mellitus	0.93 (0.83–1.04)	–
Coronary artery disease	0.90 (0.80–1.02)	–
Congestive heart failure	0.71 (0.62–0.81)	0.84 (0.73–0.98)
Cancer	1.28 (1.12–1.45)	1.65 (1.40–1.95)
Renal disease	0.71 (0.59–0.86)	0.98 (0.79–1.21)
Venous thromboembolism	1.62 (1.29–2.04)	1.64 (1.30–2.07)

CI = Confidence interval; CTAS = Canadian triage acuity score; EMS: Emergency Medical Services; OR = Odds ratio.

## Discussion

Using linked administrative databases, we explored acute COPD presentations to EDs across one province in Canada with special emphasis on the investigative approach ordered during these important encounters within the Canadian health care system. Overall, nearly one in six patients with AECOPD as a primary or secondary ED diagnosis received some form of investigation for PE. By far, the most common test was a blood d-dimer. Despite the frequency of testing, the prevalence of PE in the ED or after hospitalization was lower than reported elsewhere [7]. Among studies included in a systematic review on the topic, only one included patients from the ED and found a similarly low prevalence of PE during AECOPD (3.3%) [8].

It may seem surprising that D-dimers were drawn and, even when positive, subsequent advanced imaging was not performed. Clinicians are not obligated to “chase” D-dimer results and an elevated D-dimer in the setting of AECOPD may reflect systemic inflammation and infection in the chest cavity. Importantly, minimal elevations were often negated by using an age-adjusted cut-off, which may have been used in this setting. Moreover, many patients underwent CTPA without D-dimer testing and even when D-dimer testing was positive (either by conventional or AADD cut-offs), the results were apparently ignored, since further advanced imaging was not ordered.

One of the unique findings in this study was the granular description of ED times. First, the PIA values fail to provide evidence that crowding metrics were factors influencing the delays observed in the ED visit. Second, the proportion of patients who were designated for admission, regardless of investigations for PE, were similar. Third, the investigation of PE in the ED added nearly 2 hours on to an ED visit, appeared to contribute to ED crowding and to delay departure of patients to their place of residence and comfort. Another unique feature of this study was the comprehensive follow-up and outcome assessment. For example, regardless of whether patients received investigations for PE or not, both groups had similar returns to ED at 30- and 180-days, death within 30 days (for all causes) and death within 30 days of discharge.

In addition, the overall prevalence of PE among patients with AECOPD presenting to the ED was higher in patients with a positive D-dimer. Despite significantly higher proportion of patients undergoing advanced imaging for PE, the results suggest that using an AADD cutoff and considering other patient factors might be cost-effective and safe in patients with low probability of PE, preventing unnecessary advanced imaging in approximately 13%.

To understand the clinical practice, we explored factors associated with PE investigations through adjusted analyses. Reassuringly, some factors readily available at the ED presentation such as existing conditions (e.g., cancer), severity indicators (e.g., CTAS score of 1 or 2), and specific presenting complaints (e.g., chest pain and shortness of breath) were significantly associated with investigations for PE. Moreover, there were some factors such as increasing age, existing conditions (e.g., CHF), severity indicators (e.g., CTAS score of 4 or 5), and specific presenting complaints (e.g., cough) that were negatively associated with investigations for PE. Finally, there were perplexing results whereby arrival in evening hours and mode of arrival via EMS were negatively associated with investigations for PE. Overall, these results don’t entirely explain the variation observed.

Concerted efforts have been made through international organizations such as Choosing Wisely to limit the use of unnecessary testing, procedures and/or medications in health care [15]. Many tests are performed by clinicians for fear of missing a diagnosis, medico-legal concerns, and patient expectations. Repeated studies have identified excessive testing for PE in EDs, and calls have been made to adopt a more evidence-based approach [9]. Despite the frequency of testing for venous thromboembolism (17%) as a cause of the exacerbation in this study, the event rates in our and another ED study were lower than expected. Moreover, it appears that testing doesn’t influence important post-ED outcomes such as re-visits and mortality. Since PE is a serious condition, AECOPD and PE share similar, albeit not identical, features (e.g., dyspnea), and misdiagnosis of PE is well known, we conclude that rather than dismiss the diagnosis, clinicians should adopt a more rational approach to investigation. Fortuitously, this approach has recently been endorsed by investigators who developed and validated the use of clinical signs (non-purulent acute exacerbation of COPD, evidence of DVT and likely diagnosis of PE) and D-dimer measurement for the diagnosis of PE in a hospitalized cohort of patients with COPD [16].

There are many reasons why clinicians should be concerned with the issue of extra testing. First, advanced imaging delays departure from the ED by approximately two hours. This adds to the burden of ED crowding and opportunity costs. Second, unnecessary testing increases costs to the health care system. Third, excessive testing can increase the risk of false-positive results, which have their own consequences (e.g., anticoagulation risks, insurance issues for patients)**.** Fourth, while patients receiving CT scans are being subjected to needless radiation exposure, many of these patients present repeatedly as their disease progresses and will also be receiving cumulative radiation exposure.

Overall, the decision to investigate a patient for PE is complex and multi-factorial; more rational approaches to investigating PE appear urgently needed. Recent efforts show promise [16] and advances in artificial intelligence/machine learning have the potential to contribute to ED decision-making in the futur [17].

## Limitations

This study has several limitations that require discussion. First, the study relied on administrative data and did not examine individual charts. Consequently, data on other behavioural factors (e.g., smoking status, activity level/exercise, diet, etc.), while often incompletely documented, were not collected. In other studies using ICD coding, however, chart review failed to identify patients with a diagnoses of PE who were missed by *ICD-10* coding [9]. If any bias exists, the use of administrative data codes likely overestimates the frequency of PE [18].

Second, we have included all J44.x codes for COPD; however, other ICD-10 codes may have been used (e.g., community acquired pneumonia, heart failure, diabetes) that reduced the number of patients with AECOPD in the cohort. For example, heart failure is a risk factor for PE and up to a third of patients ultimately diagnosed with PE are initially misdiagnosed as having an exacerbation of heart failure [19]. Third, we did not collect some specific PE risk factors (e.g., immobility, recent surgery, travel, etc.) and physical findings (e.g., leg circumference, pitting oedema, varicose veins, alternative diagnoses, etc.), since these are unavailable in the administrative databases employed. This prevented the calculation of a clinical decision rule score and pre-test probability. Moreover, it precluded the assessment of appropriateness of the investigations undertaken.

Fourth, the administrative data does not provide reasons for why clinicians ordered CTPA scans; other reasons to request advanced imaging in AECOPD include pneumonia, pleural effusions, and pneumothorax/pneumomediastinum. In these other conditions, patients generally receive non-contrast CT scans, not CTPA. In a small percentage of cases, clinicians may not have been searching for a PE; however, admission decisions may have been delayed by consulting services requesting PE exclusion/confirmation. Finally, the ED sites in this study were not using the age-adjusted D-dimer threshold at the time of the study, and these results may not apply to other centres that do. Although individual emergency physicians may have applied the age-adjusted D-dimer threshold in their clinical practice, the data collected and reported in the electronic medical system did not account for these adjustments.

Notwithstanding the above concerns, this multi-centered study used robust and valid linked administrative databases to study a large Canadian patient sample that were typical for AECOPD presentations. The linkage to laboratory and imaging databases enhanced our confidence tin the findings.

## Conclusion

Although one in six patients with AECOPD were investigated for PE in these Canadian EDs, the overall prevalence of PE was less than 1% in this cohort. Despite a high proportion of patients undergoing advanced imaging for PE, the results suggest that employing pathways using valid CDRs that provide pre-test probabilities, selective investigations (e.g., AADD for PE) and information readily available at presentation might be a more cost-effective and safer strategy to prevent unnecessary imaging, especially in patients with low probability of PE. These study findings will also provide clinicians with more valid and robust evidence upon which to base shared decision-making in the acute setting.
